# Foreign

**Published:** 1841-01-09

**Authors:** 


### FOREIGN.

*Lectures on Amputation and on the Nature, Progress, and Terminations of the Injuries for*

*which it is required.* By RUTHERFORD ALCOCK, K. C. T., &c., late Dep.-Insp.-General of Hospital with the Auxiliary Forces of Portugal and Spain.—Three years ago I submitted to the profession, in a very curtailed form, some views on the questions involved in the subject of amputation, and the injuries for which its performance is required.* The opinions given were drawn from the experience of many years active service, during which large hospitals were under my sole direction and control, and sick and wounded in large numbers constantly under my observation. The principal facts and views contained in that work, found a place at considerable length in the pages of the Lancet; and they have since been very frequently referred to and quoted in various publications, periodical and others, as meriting some attention. In the very able "Retrospective Address in Surgery, delivered in July, 1839, before the Meeting of the Provincial Medical Association at Liverpool, by J. P. James, Esq., Surgeon to the Devon and Exeter Hospital," I find my opinions and statements are referred to in a flattering manner: and I understand they have also found a place in the Retrospective Address of Mr. Dodd, of Chichester, delivered this year at Southampton.

Even had I not entertained the intention, when I first published my little work, of giving more at length and in detail the conclusions—then only very briefly stated—with all the facts upon which they are based, I should have found sufficient inducement, in the favourable notice the profession still bestows upon my past labours, to do so now.

The concluding lines, however, of that work pledged me to undertake, at some future day, the task I have now completed. It has required much time and labour, by analysis and classification of cases in numerous series, to demonstrate to others the correctness of many views, at variance occasionally with those most generally received, but which I, nevertheless, felt firmly convinced were both true and important.

It was necessary to arrange each series of cases so as to show distinctly their bearing not only upon my own views and conclusions, but upon the opinions and principles of practice advocated by those who have taken a prominent part in the attempts of the profession to establish the principles that should guide us, on correct and unalterable bases. In reference to injuries and diseased actions which require and warrant amputation—the period best fitted for the operation, the supervening actions which endanger or destroy life under the great variety of circumstances arising from degrees and kinds of injury—different modes and periods of operation—differences of temperament, age, climate—of external and collateral circumstances—these are all required to be kept in view as objects worthy of investigation, and requiring to be tested and proved.

It would be out of place in a series of lectures devoted to investigations and objects of a strictly practical nature, to trace back the history of amputation, or to show what the opinions of surgeons were on the subject under consideration, a century ago. My duty is to bring before the profession the opinions of the present day—taught and acted upon as established principles of practice—to inquire how far they are founded upon correct premises—with what errors they are chargeable—whether these extend to the *conclusions* on which modern practice is founded. Finally, to trace the ultimate consequences of these conclusions upon the various classes of amputations, and severe injuries or diseases affecting the health and safety of the limb in the first instance, and subsequently the life of the patient.

It may appear to some, on glancing at the title of these lectures, that to write on the subject of amputation—on the relative value of primary and secondary operations, &c., is "*precher a la conversion;*" that the questions have been long and definitively settled, the injuries for which amputation should be employed fully determined, &c.; that, in a word, any farther facts, opinions, or arguments on these and all other points connected with amputations are superfluous, and the labour a work of supererogation.

I say this may appear to others, for I went to the field myself, fully relying upon the "settled questions," determined to have no secondary amputations if I could avoid it, and no delay in operating on cases where I could not entertain a well-founded hope of saving a useful limb. With such strong prepossessions and firm belief in what I had been taught in the schools, I entered upon my responsible duties. It was not long before many results first surprised and then startled me, creating very serious doubts in my mind. Primary operations, followed by death, which, according to my guides, should have lived; secondary amputations giving cases of recovery under the most disheartening circumstances. I felt it absolutely necessary to penetrate the cause of these seeming contradictions, which unsettled all my previously-formed convictions. In labouring to dissipate them by careful observation where opportunities were abundant, at the bedside, and subsequently by reflection, searching either to confirm the truth and correctness of the premises and conclusions on which I had previously acted, or prove them to be erroneous, I satisfied my own mind at last. It may be useful to anticipate similar doubts in the minds of those who may have to decide the questions *practically*, as I had, for the lives and limbs of their fellow-creatures; the doubts and the grounds for them; the series of facts, and the reasoning which wrought in me conviction, I propose to bring forward in these lectures; con-

* Notes on the Medical History and Statistics of the British Legion in Spain. Churchill, 1838.

vinced that by facts most carefully sifted and by reasoning, should the questions incident to the subject of amputation be decided; and not by rapid generalizations, loose analogies, and unhesitating assertions or opinions. In the progress of my inquiries, the first satisfactory conclusions obtained, though tardy, were of a nature to lead rapidly to many others; viz. 1. Many of the leading conclusions on which authors had framed the rule of practice were drawn from false premises, and proved occasionally to be as erroneous as the data. 2. That even where the conclusions were correct, they were often so by mistake, if I may be allowed the expression; that is, they were illogical, and in reality inconsistent with the premises on which they were supposed to be founded. 3. That the advocates of each set of opinions frequently contradicted themselves, and even others. These statements will be reproduced in detail, and amply borne out, by individual facts as by general results, in the course of these lectures.

The various questions connected with amputation, so long agitated, so warmly discussed, for more than half a century, are yet to settle definitively, and the present observations are offered as contributions towards the effecting so desirable an object. Although Sir George Ballinghall, in his last edition (in 1838) of a valuable work on Military Surgery, seems to consider all the more important questions finally determined, yet he passes in review many points on which he cites opposite opinions, and others where he specifies a want of sufficient evidence. Thus, p. 368, "I know of no comparative estimate of the results of amputations performed by the circular incision and by the double flap, which will enable us to decide their respective merits by the test of experience." This is the latest writer on the subject.

The general tendency of very recent writers, such as Gendron, Hayward, Norris, and others, who have given opinions and the results of their experience to the profession, is in direct opposition to the doctrines taught by the majority of surgeons in the French and English armies, at the close of the great continental wars in 1815. Even at that period, Guthrie, Hennen, and Thompson, who gave the results, and may be considered in some sense as the medical historians of the British army-practice; Mr. Hutchinson and Sir Stephen Hammick, in a similar sense, of the navy; and Barons Larrey and Percy, of the French army, together with several authors of more fugitive productions in both countries, differed essentially; although the general purport of their labours presented something of unanimity in reference to the advantage of the primary period for amputation; yet they widely diverge from each other, sometimes as to premises, and at others in conclusions. The whole of these writers succeeding Vaure, Le Conte, and John Hunter, who had maintained opposite doctrines.

The tendency of most of the writers for the last few years, as I have stated, drawing their facts chiefly from civil hospitals, is again to support the views, to a certain extent at least, of John Hunter, and others of his period.

This glance, I think, may suffice to show, that neither the one set of doctrines nor the other have yet been based upon irrefragable evidence; and, indeed, in reference to many points, we have the opinion of the writers that there was a want of data. Dr. Thomson, who may be considered, in his observations on some six thousand wounded, resulting from the battle of Waterloo, to give the last results of British military practice, although by *opinion*, he confirms the same general tendencies, records a fact subversive of our confidence in the only numerical results on which the opinion was founded. He says, p. 226, "The results of the amputations performed in Belgium might, on the whole be said to be successful, though it certainly was not equally so with that which is stated by M. Larrey and Mr. Guthrie to have been obtained in some other countries; and what *is curious, this comparative want of success was more remarkable in the results of primary* than of the secondary amputations."

Again, p. 241, "It may be doubted whether the practice of immediate amputation would be proper or necessary in all these cases, could the wounded be conveyed directly into convenient hospitals, in which they might remain during the period necessary for their recovery; *for we have no data by which it is possible for us to judge very accurately what proportion of them would recover without amputation; how many would require amputation at a late period, and of those in which recovery should take place; in what proportion the limbs would be useful, or remain useless and troublesome.*" In reference to fractures of the thigh, also, p. 249, "A series of observations, much more extensive than any we yet possess, will be required, in order to enable us to determine what is the usual proportion of those who recover from fractures of the thigh-bone in its different parts by musket-bullets, and of those recovering who have suffered gun-shot fractures of the thigh-bone."

But independent of these admissions of the want of correct and accurately-classified data, by the chief writers of the year 1815, who, coming after Hunter, Percy, and Lombard, directed their whole efforts to refute the doctrines taught by the latter, the inconsistencies and contradictions evident in the premises and conclusions of the various writers of that period, who undertook to prove their predecessors entirely wrong, are in themselves sufficient to render it clear that, although they each may ultimately come to nearly somewhat similar conclusions, yet there are contradictions which must go far to neutralise or nullify the otherwise convincing unanimity of decision.

One great error runs through the discussion maintained by the advocates for delayed amputation on the one hand, and the later writers who reprobated such practice on the other; and as they are both sufficiently obvious, implicit faith could not be placed on the opinions of either party by any impartial observer.

While Vaure, Le Conte, Hunter, &c., insisted upon the fatal consequences of a second shock to the system by amputation immediately after an injury, exaggerating, or too widely generalising the effects immediate and remote, they partially forgot the inevitable evil consequences, of a limb irremediably injured, remaining attached to the body, and provoking the most fatal actions, inflammatory and sympathetic. The other party who succeeded them, Larrey, Guthrie, Huchinson, &c., erred not less by denying or overlooking the fact, that there *is* a second shock occasioned by amputation, and that it does and must increase the immediate chances of death, and substitute one train of accidents for another. Thus De la Martiniere says, " L'amputation faite a propos ne peut pas être regardée comme une enterprise téméraire qui ajouteroit de nouvelles sources d'accidens a ceux qui tourmentent les blessés puisque *on ne fait que substituer une plage aussi simple qu'il est facile de la procurer*." Not a word of any shock or danger attending the amputation! Again, Mr. Guthrie, following in the same track, says, " Instead, then, of inflicting an additional injury on the original one, and increasing the general symptoms of irritation in those persons with extensively lacerated and complicated wounds, they were completely relieved, became calm, tranquil in mind and body," &c.

Whether amputation quickly following a wound *increase* the general symptoms of irritation, or not, may be a question; probably, in the worst kind of cases, it may not increase them; for the injury itself would produce the worst; and, moreover, the very shock itself will often act by exhaustion as a sedative. But, even in these cases, if it do not increase them, certainly very often it does not prevent the development of an irritative fever, and symptoms, and effects, apparently depending on the double shock to the nervous system, which are fatal. It cannot seriously be disputed, that amputation *is an additional injury and a shock*, not inferior, in many instances, to the original one inflicted by the injury; and that it is impossible to "*substituer une plage aussi simple*," without causing a violent and dangerous shock.

Mr. Guthrie himself, in another page, says, "I allow amputation to be a violence superadded to the injury—a violence that occasionally destroys the patient. But it as frequently does so after secondary as primary." It is equally capable of demonstration, that there is a train of dangerous symptoms attributable to the shocks of this operation—where no other injury or shock had been received—however easily and simply the clean incised wound may be *substituted* for a lacerated limb. How is it possible to rely upon the conclusions of partisans, so eagerly bent on proving opposite principles, that they each overlook most essential and important features, or overlook at one moment what they bring forward at another.

But they are not even consistent, for De la Martiniere, alluding to the prevalent cause of ill success in primary amputations, seeks to explain it in part by means subversive of his previous opinion—he says it is to be attributed, " a la peu abondance des forces des blesses, aux dispositions inflammatoires, a *l'irritation du genre nerveux;*" and he proceeds to add, that in effect we do observe them when amputation is performed at a much later period, when the system has been reduced, &c., the patient is *less liable to these perilous accidents*—that is, when a single shock is only suffered instead of a double one. If we turn, however, to Larrey on the same side, he gives, as the result of his latest experience, viz., of the wounded of July, 1830, and as confirming all his former opinions, the statement, that " secondary amputations have generally been followed by violent *orages.*" Both cannot be correct for they are diametrically opposed to each other.

Boucher, again, the refuter of the opinions of M. Vaure, speaking of M. Vaure's success in ten different amputations, says, it only proves that the state of weakness which is not the result of deterioration of the solids and vitiation of the fluids, as it frequently is in such cases, *is more favourable to amputation* than a state of greater vigour—the very point M. Vaure maintained.

Mr. Guthrie, in speaking of the same subject, argues that the reaction of the constitution producing high inflammatory fever, can be more readily suppressed, and with more safety to health, than an irritable constitution; he contradicts Boucher, and at the same time seems to forget altogether the fact about stated inferences as a matter of course, that in any actions supervening on secondary amputation we have to struggle against an " irritability of constitution." But there lies the question—are all cases reduced by discharge, irritable? Certainly not; and here is a proof of contradiction between two advocates of the same general conclusions, and of logical deduction, from what I must believe to be erroneous premises. Instances such as these are most frequent throughout the writings on this subject. Mr. Guthrie concludes by saying, " that as the military surgeons of Mr. Hunter's time supported one side of the question, and the military surgeons of 1815 supported the opposite, one party must certainly be in error." This seems very undeniable; but, nevertheless, the truism is more apparent than real. It seems to me, and I trust to be able to prove, that the conclusions of both parties are frequently based upon false premises; while at other times very opposite conclusions are drawn from the same data: that enlightened by some experience, and with a mind duly prepared, no one can proceed to analyse the opinions supported by the two parties, without perceiving that erroneous data, arguments, and conclusions abound in both; that sometimes the two parties nearly agree, though from apparently dissimilar premises, while they as widely differ in other instances from the consideration of similar facts; that each party abounds in contradictions and inconsistencies of the individuals with each other, agreeing, even occasionally, with few opinions sustained by their adversaries. Thus, to take the works and data furnished by all sides, it would be impossible to form anew a natural division, according to the opinions, into two opposing classes of authors. In some instances, the nominal advocates for delayed amputation will be found supporting opinions, and bringing forward facts, which, duly estimated, are strong evidences in favour of primary, and *vice versa.* Where so much of truth and error seems to abound with contradictions and inconsistencies, it cannot be matter of surprise that, notwithstanding the results of the last continental war have been held by superficial inquirers to have settled the question between the advocates of opposite opinions in relation to amputation, the question should still be perpetually recurring in practice; these very inconsistencies, errors and contradictions, becoming evident at the bed-side, and destroying all confidence in the most stoutly maintained opinions of either side.

If we required any proof of this feeling, as a general result of practice, and the study of the facts and opinions of these writers on both sides, we shall find it in the general tenor of the majority of the contributions to our knowledge of the history and progress of amputations which have appeared within the last ten years in Germany, in France, in America, and in England. The tendency of all, with few exceptions, is to re-open the discussion, expressing doubts of the correctness of the prevailing opinions established by the writers of 1815, and showing a disposition to return rather to the convictions of the authors preceding those, viz., of John Hunter's time, the purport of whose opinions were held to be diametrically opposed. This assumption, however, being only partially correct, for many of the points which have been most strenuously maintained by the former, were already conceded by the latter.

Enough, I think, has been shown to prove that the endeavour, on my part, to analyse the sources of this confusion in premises and conclusions, and by the aid of a new series of observations in the field and in the hospital, to point out the inconsistencies and contradictions, eliminate errors, and draw from correct premises the legitimate conclusions that should form our guides in practice, is not a work of supererogation. In any set of principles, whenever an inconsistency or a contradiction appears, its tendency must be to unsettle the conviction on which we had previously relied. Unless the conclusions affecting the questions of amputation are placed on proper bases, they will always be liable to change and subversion, even though they may be essentially correct; any errors, founded either in the premises or deductions, must necessarily tend to unsettle opinions on this momentous subject, and give rise to a vacillating, uncertain, and erroneous practice.

Many of the data which writers had indicated, as wanting to enable them to form an accurate judgment on some points of practical importance, I hope to be enabled to supply. The comparative results of amputations (at the three different periods, first defined by Boucher) in *civil* hospitals, for injuries resulting from the accidents of civil life; and these, again, compared with the results of amputation for chronic diseases, have never been, so far as I know, more than guessed at. This is a desideratum, I trust, also, to accomplish, to a certain extent at least.

No question of greater importance ever comes before the surgeon, and, in a military practice, none more frequently, than that of the propriety of attempting to save a lacerated and fractured limb; or the necessity, on the other hand, of at once removing it.

It is not a mere question of limb, but of human life and suffering. To attempt to save a limb when useful cure is hopeless, in a large proportion of cases ends in the death of the patient, after weeks, or months, and occasionally years, of fruitless pain and misery. To amputate, where by more judicious surgery an useful member might be saved, is to inflict a grievous and unnecessary loss upon the patient, besides subjecting him to the perils of an amputation.

More need scarcely be said, to show how interesting is the field of surgery,—how important the results, and especially how valuable are accurately-recorded facts on which we can rely, to form a judgment of the nature, progress, and gravity of the supervening actions, on amputation performed at different periods,—for different kinds and degrees of injury or disease; and these, again, under different external and collateral circumstances.

That such data have long formed a desideratum, and the want of them been often experienced, the very cursory glance already made through the records of military surgery, have shown.

Nor can it be matter of suprise, that even the late continental wars should not have enabled the respective medical staff to furnish these data. Something more than zeal and talent on the part of the medical staffs,—than many battles and their proportionate number of cases,—nay, something more even than commodious and well-regulated hospitals, is required to render the collection and accurate arrangement of such facts, in a complete form, possible. It is necessary that when the wounded are received, they should remain till the result of the cases is established, under the direction and observation of the same medical officer. It is not less necessary that the zeal and number of the medical staff, at the chief officer's disposal, should be adequate to the daily record of detailed notes in every case presenting features of interest, as regards the individual, or as forming one, however uninteresting in itself, of a class. At the same time, the number under treatment at one time, should not be so great as to prevent the superior medical officer comparing and superintending notes and cases; thus becoming responsible for the correctness of the former, and able to speak of all with the weight and the conviction of personal observation.

It may be said, and indeed it was urged by one of my critics, in 1838, when I pointed out the importance of these conditions, that such a combination of favourable circumstances for study and observation could never be obtained. I have it at heart, to prove that I contemplated no impossibility. That such opportunities must be rare, I am ready to admit; and most rare, when the war is carried on by the largest armies, and over the greatest extent of country; for under these circumstances, the constant change of wounded from one station to another, the fearful influx of numbers at particular periods, all contribute to render the attempt to record facts, in complete series, impossible.

Such opportunities, in truth, can only occur when an army is in lines, defending them for a considerable period against repeated attacks, and the hospitals in permanent stations near at hand, or in a beleaguered city. In seven years' active service in the field, there is only one period extending through any length of time, where I was placed in a condition to collect, with the necessary accuracy and completeness of detail, *whole classes and consecutive series of cases*, although I had previously been nearly a year in a besieged city. In Oporto, however, some of the conditions were wanting, and thus, to a certain extent, defeated my efforts. The facts of interest collected in the hospitals under my charge at that time, are indeed numerous; yet, with the exception of one or two of the more important classes, of which amputation is one, I am not sufficiently assured of their including all the cases to venture to make use of them otherwise than in an isolated form.

If a series be given, and there be one omission, the result is incorrect. With this truth before me, I have taken care, in the statistical returns constructed for these lectures, to confine the number of fractures, involving and not involving articulations, which were submitted to treatment and not amputated, to those which occurred at San Sebastian, in the years 1836–7. The whole of such cases are thus included, which resulted from all the actions fought within a given period in the vicinity of the lines formed to cover that fortress.

These cases were all received into the Hospital of San Telmo, of six hundred beds, treated under my own personal direction the whole period, with a numerous, efficient, and zealous medical staff, to carry out my views. Most of the actions were fought within a league of the hospital, the greatest distance three leagues, and the wounded were generally received within a few hours after they had been injured and there retained. The hospital was vast, well ventilated, and admirably situated, supplied with good diet, medicines, and all essential requisites for treatment.

In reference to the amputations, however, these, forming a class of their own, extend over many additional periods, wherever I could feel assured that my records comprehended the whole number of any given series. Thus, the statistical returns to which I shall direct attention in these papers, which give some of the results of my own practice, include one hundred and seven amputations; a number, it may be urged, too small to determine any important question conclusively in the minds of the profession. It may be so; but having watched not the progress of that number only, but more than two hundred in addition, and obtained convictions which I find are confirmed by the results of these series, each complete, I cannot but think views so supported may be worthy of attention. The amputations comprised in the tables include all arising from the British wounded in

Nine attacks and sorties at Oporto;
One action in Estremadura;
Nine in the North of Spain;

And there is one circumstance attending these cases which adds to their value,—all the wounded of each series were under the same general conditions of distance from the hospital, mode of transport, under the same general principles of treatment, and for the most part under the same roof; the majority natives of these islands, and ranging about the same age —from 20 to 30 years of age, artisans and peasants—all, therefore, are analogous in many essential features.

| | No. of Cases. | No. of Amput. |
|---|---|---|
| 1. Gunshot fractures, not implicating joints, | 167 | 60 |
| 2. Ditto, involving the articulations, | 97 | 49 |
| 3. Cases of spontaneous disease leading to amputation, | 8 | 8 |
| | 272 | 117 |

The total number of cases, therefore, is 272, the amputations amounting to 117, upon which are founded the various conclusions. The two first classes are exclusively the casualties of military life. In order to complete the task I proposed for myself, I have formed two tables; one of all the amputations occurring in the Massachusetts Hospital, from its formation in 1822 to the end of '39, a period of 17 years. The second contains all the cases performed in the hospital of Pennsylvania, from Jan. 1831, to Jan. 1840. I have further separated these classes into injuries and diseases. The materials of these tables are furnished by the North American Journal for August, 1838, and May, 1840; the first by Dr. Hayward, one of the surgeons of the Massachusetts Hospital; the second by Dr. Norris, also one of the surgeons of the Pennsylvania Hospital. Dr. Norris gives us 79 cases; Dr. Hayward, 67. The returns of Dr. Norris and Dr. Hayward leave no doubt in my mind of their completeness as to numbers and accuracy. I speak of the results which they show, therefore, with the same confidence, so far as their details extend, as I do of those which I myself have recorded:—

109 Amputations for gunshot injuries, and
145 Civil injuries of civil life, or chronic diseases;

———

254 Gives a fair number on which to draw conclusions.

Of the general results furnished by Mr. Phillips, in the "Medical Gazette," they may contain complete series; but as there is no evidence that they are not imperfect in this sense, collected in fragments from many sources, I do not feel warranted in taking any of the numbers as good and sufficient evidence in deciding questions where the omission of an unit, or a single case in any series, will invalidate the total result and the legitimate conclusion. The same reasoning holds good with the large return of the British Peninsular army during the last six months of 1813, setting aside the absence of very essential details. I think it more than doubtful how far one may depend upon the *whole* that occurred over the whole breadth of Spain were included, or what modification any omissions might have occasioned.

The circumstance to which I have alluded, viz., the exceeding rareness of the opportunity of collecting such information as I have classified in a continuous and complete form, must add to the value, if the facts be shown to be accurate. And with respect to these, I may add that I have been more anxious to furnish data worthy of confidence, on which others might reason, and from which they may draw their conclusions, than to enforce the views and opinions I shall state as the result of my own mind.

The time which has elapsed since I first announced the intention I now carry into effect, is in itself a proof how serious I have considered the nature of my task, and that I shall not intrude on the profession any hastily formed conclusions, but facts carefully analysed, and opinions maturely weighed.

I have already stated the object I have in view, viz., to determine, by a consideration of all the facts connected with the subject, the true principles of practice, MILITARY and CIVIL, in all complicated injuries of the extremities, and especially in reference to amputation, the periods and modes of performance. To fix these principles on a solid basis of facts and legitimate deductions, that they may be less open to doubt, and less liable to be subverted, than those existing, because freed from their contradictions and inconsistencies; finally, to render those principles applicable to the question of amputation for chronic disease, showing their bearing upon the latter class of cases by comparative results.—*London Lancet.*

———

*Case of Abscesses occurring in the Brain.* By R. BOYD, M. D., Resident Physician.—Mary S., aged nineteen, admitted into the St. Marylebone Infirmary, in the last stage of phthisis, states, that she has been subject to spitting of blood, on making any exertion, for the last eleven years, and has been getting gradually worse for the last three months; the spitting of blood during the last week amounted to about three pints. Her mother died under my care of phthisis about three years ago, at which period this girl had medicine prescribed for her, of which, or of her symptoms, no note was taken at the time. I am indebted to Dr. Harrison (whose patient she was in the infirmary) for the following brief note of her symptoms on admission:—Pulse rather quick, jerking, compressible; skin hot; tongue whitish; bowels open, though usually confined; catamenia ceased four months ago; severe headach during the last three days; nausea for the last week; vomited this morning some greenish-coloured matter. On examining the chest, a dull sound was elicited by percussion under the right clavicle, with considerable flattening of that region, &c. During the two following days the headach became progressively worse; considerable excitement now existed, and she often shrieked out from the intensity of the pain; the vomiting still continued; pupils slightly dilated; following day more composed; pain less acute; seemed afraid to move her head from the pillow; spoke in an under tone. The next morning little apparent change; she could get out of bed without any assistance when she had occasion to do so. About half an hour after this she died quite tranquilly.

*Autopsy twenty-five hours after death.*—Head only to be examined. On removing the dura mater slight cohesion was found to exist betwixt the surfaces of the arachnoid, which presented a dull, glazed appearance; it communicated to the touch a feeling of dryness, with unctuosity; the convolutions appeared flattened; veins considerably distended with blood. On the superior, posterior, and outer portion of the left hemisphere was observed a deposit of purulent matter, as large as a shilling, extending into the substance of the brain; the anterior half of the body of the lateral ventricle was filled with pus, streaked with blood. On examination a deposit about the same size as the first was found in the inferior and inner portion of the corpus striatum; a smaller deposit existed in the inferior, anterior, and outer portion of the left hemisphere; and a fourth, still smaller, was found in the cortical substance of the upper and anterior portion of the right hemisphere. On the surface of the arachnoid, at the junction of the pons Varolii, with the medulla oblongata, there was a yellowish deposit, probably the result of inflammatory action. The interior of each of the cavities containing the purulent deposits seemed to be lined with a distinct membrane; that lining the small cavity in the cortical substance was smoother than those of the others, which were all more or less irregular in their interior, especially that in the corpus striatum, on the inferior surface of which there were some enlarged vessels, and the pus in this situation was mixed with a little blood. The matter in all the cavities, on examination, presented the characters of healthy pus.

The symptoms of cerebral disturbance were only complained of eight days before death, and they did not arrive at their height till forty-eight hours before that event, when she presented all the symptoms of a person labouring under acute arachnitis. I recollect a male insane patient under the care of the late Dr. Sims, exhibiting very similar symptoms, followed by death; he said, at the time, we should probably find a dryness of the arachnoid membrane. The post-mortem examination proved that his opinion was correct. The late Dr. Hooper of this institution has recorded a few cases of a similar nature. The works of Morgagni, Portal, Baillie, Abercrombie, &c., furnish several examples. A case occurred in the practice of this infirmary of abscess in the pons Varolii, which was exhibited by Mr. Obre at one of the early meetings of the Pathological Society last season. *Vide Lancet*, March 28, 1840. Such cases are, notwithstanding, very rare, only two or three examples of it having occurred out of many hundreds of examinations of cerebral complications made at this infirmary during the last six years. Cerebral symptoms, at the termination of phthisis, are by no means uncommon; from an analysis of fifteen cases of that disease which were examined here during the autumn of 1839, it appears two cases were attended with delirium: in one of these there was softening of the septum lucidum, and about three ounces of fluid in the lateral ventricles; the other case also contained a somewhat smaller quantity of fluid in the lateral ventricles. Dr. Clendinning, in one of his clinical lectures here last year, related the case of a young man who was admitted into the infirmary in a state of delirium, and complaining, by various intelligible gestures and cries, of intense pain of the head, without coma or affection of the pupils, and making no other complaint, nor seeming to suffer at all otherwise. He was wasted, and had a slight cough, without expectoration; dulness, on percussion, and depression of both subclavian regions. The restlessness of the patient prevented satisfactory auscultation, but the case needed no further light than that elicited by the fingers for decisive diagnosis. After ten days or a fortnight he sank. The brain and membranes were congested, and pure serum, amounting to above two ounces, existed in the ventricles; the convolutions moderately flattened. There was no pus, or lymph, or thickening, or other unequivocal evidence of inflammation in the head. Large excavations existed in the apices of the lungs. The closing scene of other chronic diseases are also occasionally marked by delirious excitement, resembling that of arachnitis: it often happens, in such cases, that we are unable to detect any alteration of structure, and have to rest satisfied with denominating it a functional derangement.—*London Lancet.*

---

*Case of Varicose Aneurism terminating fatally.*—A peasant, aged forty, was admitted into hospital on the 9th of December, with a varicose aneurism at the bend of the left arm, which had appeared a few days after venesection. A compressive bandage was applied for twenty-two days round the whole arm without advantage. On the 1st of January the brachial artery was tied according to Anel's method, and the operation was followed by the ordinary symptoms until the fifth day. At this period gastric symptoms set in; and the arm swelled to such a degree, that the aneurismal tumour, which was as large as a hen's egg, could no longer be distinguished. The wound began to suppurate copiously; and an obstinate diarrhœa terminated the sufferings of the patient on the 21st of January.

*Dissection.*—The brain, lungs, and intestines, showed traces of inflammation and accumula tion of purulent motion. The tied artery had suppurated, both above and below the ligature. The coagulum was of a conic shape; and close to it, as is usual in such cases, lay the sac of the aneurism. When opened, it displayed a double cavity, which appeared to have been formed at the expense of the cellular substance situated between the artery and the vein.—*Zeitschrift für die gesammte Medecin, from the Jornal da Sociedade das Sciencias medicas de Lisboa.*

---

Both to Blame.—'Tis a pleasant and witty saying that Plutarch says Democritus was wont to have, viz. "that if the body and soul were to sue one another for damages, 'twould be a question whether the landlord or the guest were most faulty."

